# A New Wireless Biosensor for Intra-Vaginal Temperature Monitoring

**DOI:** 10.3390/s101110314

**Published:** 2010-11-17

**Authors:** João M. L. P. Caldeira, Joel J. P. C. Rodrigues, João F. R. Garcia, Isabel de la Torre

**Affiliations:** 1 Instituto de Telecomunicações, University of Beira Interior, Rua Marquês D’Ávila e Bolama, 6201-001 Covilhã, Portugal; E-Mails: jcaldeira@it.ubi.pt (J.M.L.P.C.); jgarcia@it.ubi.pt (J.F.R.G.); 2 EST—Polytechnic Institute of Castelo Branco, Av. do Empresário, 6000-767 Castelo Branco, Portugal; 3 E.T.S. Ingenieros de Telecomunicación, University of Valladolid, Paseo de Belén 15, 47011 Valladolid, Spain; E-Mail: isator@tel.uva.es (I.T.)

**Keywords:** e-health, IEEE 802.14.5, temperature, Wireless Body Sensor, Wireless Sensor Networks (WSN)

## Abstract

Wireless Body Sensors for medical purposes offer valuable contributions to improve patients’ healthcare, including diagnosis and/or therapeutics monitoring. Body temperature is a crucial parameter in healthcare diagnosis. In gynecology and obstetrics it is measured at the skin’s surface, which is very influenced by the environment. This paper proposes a new intra-body sensor for long-term intra-vaginal temperature collection. The embedded IEEE 802.15.4 communication module allows the integration of this sensor in a Wireless Sensor Network (WSN) for remote data access and monitoring. We present the sensor architecture, the construction of the corresponding testbed, and its performance evaluation. This sensor may be used in different medical applications, including preterm labor prevention and fertility and ovulation period detection. The features of the constructed testbed were validated in laboratory tests verifying its accuracy and performance.

## 1. Introduction

Applications of sensor networks have evolved in many fields of investigation field due to their large applicability and development possibilities, especially in the Wireless Sensor Networks (WSN) area. Low power consumption and low data rates are the most important features for WSN applications. A WSN consists of a group of sensors that monitors some physical or environmental parameters. Within a WSN there exist three fundamental agents: the sensor node, the event, and the reporter. The sensor node captures the event parameter, reads, and sends the information to be studied by the reporter [1,2]. The reporter is the final user who will analyze and try to get solutions.

Nowadays, due to the technological evolution of e-health applications it is possible to have sensors of all the sizes and with numerous features, even sensors that can be placed inside (intra-body sensor) or outside (inter-body sensor) the human body, typically in contact with the skin. All these types of sensors must deal with many constraints on resources such as energy, memory, computational speed and bandwidth. Sensor networks could be applied in the medical environment, helping with the gathering of data for fast diagnoses and providing monitoring services [3]. The concept of “continuity care” has been increasingly adopted by the health community. These kinds of applications have experienced considerable growth, which contributes to the improvement of human life conditions and helps the progress of Medicine by improving disease diagnoses. In this context, a Body Sensor Network (BSN) is a sensor network for body applications. These sensor networks are applied in medical care and biofeedback, providing healthcare monitoring services [3]. The aim of BSNs is to provide continuous monitoring of patients in their natural physiological state so that transient but life threatening abnormalities can be detected or predicted. This network is composed of a sensing node with a processing unit and a limited power supply. If the sensing node is provided with a wireless transceiver we are then dealing with a WSN [2]. In Body Area Sensor Networks (BASNs), the signals collected by sensors relay them to the sink node and are connected to a central computer [4–6]. The communications between sensor nodes usually employ wireless technologies like Bluetooth and Zigbee [7] over IEEE 802.15.4, but the most used and best one for wearable health applications is the Zigbee communication protocol due to its lower power consumption [8,9].

Most studies based on body temperature control started initially as a tool to detect the female fertility period by observing the increase of body temperature, know as Basal Body Temperature (BBT), taken with a basal digital thermometer [3]. This method is very painful for females and does not guarantee the validity of the gathered data. Many reasons could account for the shortcomings of this method, like equipment accuracy, appropriate environment, and external temperature factors or misuse of the equipment.

All of these factors, plus the reduced number of biosensors available on the market to perform this task, justify our effort to develop and implement this type of biosensor. Another motivation stems from the planned close collaboration with a medical team, which provides great confidence for all stages of the development. This work will provide a great deal of temperature and acquisition time (day and hour) data that could be very useful for medical studies. A team of physicians from the Health Sciences Faculty, University of Beira Interior (Covilhã, Portugal) will study a possible correlation between the intra-vaginal temperature and different stages in the female reproductive process in order to prevent (or promote) pregnancy issues. This closed collaboration is an important contribution for the medical validation of accuracy, usability, efficiency, and system performance evaluation and validation.

This paper addresses BSN issues because all the monitored parameters are directly collected from the human body. The major objective of this paper is the proposal of a new intra-body sensor for e-health applications focusing on intra-vaginal temperature monitoring, and its corresponding prototyping, performance evaluation, and validation. A detailed design of a new intra-body sensor, specifically designed for temperature monitoring with wireless communication is presented, as well as construction of a testbed and the results obtained to validate the system.

The remainder of this paper is organized as follows. Section 2 analyzes the state of the art of available systems for human body temperature monitoring. Section 3 describes the essential requirements that are featured in the new sensor device and its prototype design. Section 4 focuses on the results achieved with the created testbed and Section 5 presents a discussion and our conclusions.

## 2. Related Work

The control of intra-vaginal temperature allows the detection of several symptomatic situations in women. One of the best known is the occurrence of ovulation and fertility periods. This womens’ parameter could be used in studies of its variations and the evaluation of the effectiveness of gynecological therapeutics, to support to discovery of new contraceptive methods, in the prevention of pre-term labor and the detection of pregnancy contractions. The available solutions to evaluate the female temperature are almost all based on coetaneous temperature measurements. These body temperature values, as presented in [10], are highly dependent on the environmental temperature. Therefore, the use of women’s core-body temperature values could improve the validation of monitoring systems in detection and control of the aforementioned womans’ health situations. Over the years some systems to control and monitor women’s body temperature for fertility assistance purposes have emerged.

As proven in the AMON project [11] the correlation between coetaneous temperature and core temperature is very difficult to establish. This situation led to the use of a temperature sensor not being recommended in this project for medical purposes. The DuoFertility project [12] proposed a commercial device for continuous measurement of body temperature. It comprised three modules: a temperature sensor which is placed in the armpit, a reader unit, and the corresponding application software. A reader unit module is used to gather all the measurements collected by the sensor. This module can be attached to a computer, and the third module is an application software to graphically visualize the temperature values. This system uses the coetaneous temperature to predict the timing of the fertility period. As mentioned above, these temperature values are very dependent on the environmental temperature, so the use of these values could lead to wrong interpretations.

In [13] a method for detecting and predicting the ovulation and the fertility period in female mammals is described. This method gathers information relating to the fertility of female mammals and comprises the following steps: (*i*) taking multiple temperature readings from the female mammal during an extended period; (*ii*) identifying and disregarding temperature readings having one or more characteristics of irrelevant or faulty data; (*iii*) obtaining one or several representative temperature values for the extended period; (*iv*) repeating steps (*i*) to (*iii*) over multiple extended periods; (*v*) analyzing the representative temperature values obtained over multiple extended periods indicative or predictive of ovulation in order to provide information related to the fertility of the female mammal. This method only describes a procedure for obtaining temperature measurements for fertility purposes in female mammals and not really an actual hardware system that allows this operation.

In [14] and [15] a sensor system for intra-vaginal temperature was presented. This system is based on a thermistor unit to be placed inside a woman’s vagina. This unit was attached to a processing unit using a flexible cable. The processing unit was maintained outside the women’s body. This system obtains good results in monitoring a woman’s intra-vaginal temperature, but it is a uncomfortable to use due to the flexible cable used to interconnect the two parts of the system. Finally, Freundl *et al*. developed a new Quality Index (QI) and suggested a new method to test different cycle monitors or fertility prediction methods used to detect the fertility window for contraception [16].

The next section presents a biosensor system for intra-vaginal temperature collection. The biosensor was designed to be placed inside a woman’s vagina near the cervix. Therefore, it collects core-body temperature instead of coetaneous temperature. It has also the ability to collect long-term intra-vaginal temperature measurements using a microSD (micro Secure Digital) card.

## 3. Methods

### 3.1. System Design

Due to the working location of the proposed sensor, it has to accomodate some anatomic limitations, namely, it should be easy to place inside the vagina and comfortable, because the main focus is to help prevent problems, not cause them. Following a medical recommendation it was determined that the sensor board and other peripherals had to fit in a container of about 60 mm × 18 mm in area, so a 30 mm × 16 mm size became the target for the main board with a microcontroller, a microSD card slot, a 2.4 GHz transceiver (IEEE 802.15.4), and a computer interface included. The use of a shape similar to a simple tampon seems to be a perfect choice. It is familiar to women, easy to use, anatomically perfect, and it has an appropriate size to accommodate all the features mentioned above.

**Figure 1.**
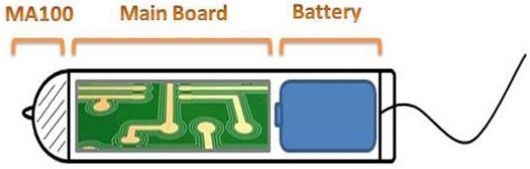
Conceptual design of the new intra-vaginal temperature monitoring sensor mote.

The tampon-like shape presented in Figure 1 shows the conceptual design of the new proposed sensor. As may be seen, the thermistor is placed on the top of the container, the electronic circuit in the body of the container, and the battery in the tail. The enclosure must fulfill strict sanitary conditions so it needs to be properly closed and avoid in any way contact with the exterior. As known, the vagina is a humid place and no fluid should make contact with the electronic part to avoid electric conductivity. The electric current range used to power this circuit is not too high, but it is enough to cause damages to sensitive and tender skin like burns or others injuries. As concluded in [17], 2.4 GHz radiation has no effect on the human body, and therefore, wireless modules enabling 2.4 GHz technology could be used safely in the construction of intra-body sensors.

With the woman’s comfort in mind, the sensor should operate when placed inside vagina. This feature avoids the need to take it out every time it is necessary to perform an operation to collect the measured temperature values. This feature will be implemented on this new sensor platform with the inclusion of a wireless communication module supporting the IEEE 802.15.4 standard. This feature also allows re-configuration of the sensor’s mode of operation without any physical connection to the programming dock/station.

**Figure 2.**
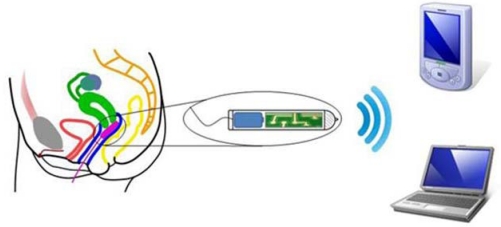
Illustration of the system architecture.

In WSNs it is expected that all nodes have a transceiver layer and a battery to achieve mobility, and spend most of the time in a low-power state, only waking up when readings and transmissions are required, as may be seen in Figure 2. The sensor is designed for collecting intra-body temperature measurements over long periods of time (e.g., during a complete menstrual cycle). This feature is guaranteed with the inclusion of a microSD slot for collection of a large amount of data. The sensor design includes a rechargeable battery with a regular voltage of 3.6 V and a capacity of 450 mAh, which can easily cover the power requirements of all the components for long periods of operation.

For temperature measurements a thermistor is included to measure the intra-vaginal temperature. The conception of this biosensor is only possible because of the use of Altium Designer, a software tool for schematic design. It can be used to design analog circuits, revise digital schematic diagrams for an existing PCB or to complete a hierarchical block design. Apart from its design functions, it also provides various built-in features for design verification and manufacturing processing. Altium Designer capture provides a component information system that allows one to identify, utilize and design with preferred parts.

### 3.2. Hardware Description

This section provides a detailed description of the features of the hardware components chosen to develop a single sensor board. Various components such as the Texas Instruments™ MSP430F2274 [18], Chipcon CC2420 [19], Antenova IMPEXA 2.4 GHz Antenna [20], TPS60100 [21] and the temperature sensor unit are described according to their contributions to the performance of the system.

The Texas Instruments™ MSP430F2*** [18] is one of the core components of the baseboard and its primary advantages are its extremely low power consumption during periods of inactivity and its proven history for medical sensing applications. The MSP430 is based upon the 16-Bit RISC CPU, peripherals and an adaptable clocking mechanism connected via a Von-Neumann memory address bus (MAB) and memory data bus (MDB). The architecture, combined with five low-power modes is optimized to achieve extended battery life in portable measurement applications. The device features a powerful 16-bit RISC CPU, 16-bit registers, and constant generators that contribute to maximum code efficiency. The digitally controlled oscillator (DCO) allows wake-up from low-power modes to active mode in less than 1 μs.

The CHIPCON CC2420 [19] is a single-chip 2.4 GHz IEEE 802.15.4 compliant RF transceiver designed for low-power and low-voltage wireless applications. The CC2420 includes a digital direct sequence spread spectrum base band modem providing a spreading gain of 9 dB and an effective data rate of 250 kbps. The CC2420 is a low-cost, highly integrated solution for robust wireless communication in the 2.4 GHz unlicensed ISM band.

To complete the IEEE 802.15.4 compliant wireless communication the Impexa 2.4 GHz SMD Antenna from ANTENOVA was the best choice. This antenna is intended for use with all kinds of 2.4 GHz applications such as in mobile phones, PDAs, PNDs, headsets, MP3s, laptops, PC-cards and sensors.

The voltage regulator chosen was the Texas Instruments TPS60100 [21]. The TPS60100 charge pump provides a regulated 3.3 V output from a 1.8 V to 3.6 V input. It delivers a maximum load current of 200 mA. Designed specifically for space-critical battery powered applications, the complete charge pump circuit requires only four external capacitors. The circuit can be optimized for highest efficiency at light loads or lowest output noise.

The thermistor electrical resistance can have a proportional (PTC type) or inverse variation (NTC type) with the increase of temperature. In the MA100 this variation is negative because it is a Negative Temperature Coefficient (NTC) type, so the resistance decreases with the increasing temperature. The NTC type of thermistor is more sensitive than other resistive sensors like Resistance Temperature Detectors (RTDs) or thermocouples, but being more sensitive means that it has a non-linear behavior and therefore a circuit is needed to adjust the exponential curve in a way that makes it approximately linear. Thermistors have a time constant which affects the time taken to make up 63% of the next temperature value. In power consumption the thermistor needs around 100 mA of current to start and power dissipation around 2 mW/°C. NTC Thermistors can have a stable acquisition in a range of −50 °C up to 150 °C.

The battery chosen is a lithium battery from GMBPower [22] measuring 15.5 mm × 13.5 mm, with a capacity of 450 mAh. This battery in particular was chosen due its circular shape and large capacity. As a regular battery, the nominal voltage will decrease as the battery is discharged. In order to provide a regular voltage of 3.3 V on the system, a voltage regulator is needed.

### 3.3. Testbed Architecture

**Figure 3.**
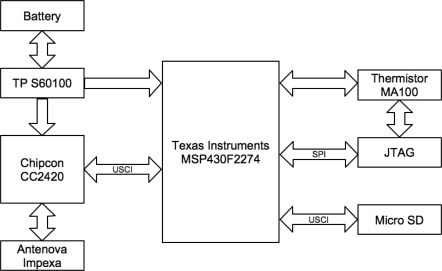
Biosensor architecture block diagram.

This sensor node requires a small size and a long lifetime in order to satisfy a large number of applications [2]. The sensor node includes a small PCB with a microcontroller, a small rechargeable battery, an external memory card slot header, and a low power radio chip. Figure 3 presents the block diagram of the architecture used in the construction of the proposed sensor testbed.

The microcontroller is the main element of the sensor because it influences the rest of the solution; a low cost microcontroller with low dynamic power consumption is essential. Thus, the Texas Instruments MSP430F1611 [23] was the best choice for this testbed because it is a component that was already used in previous work and it fulfills the most necessary requirements for the testbed construction. In addition the microcontroller selected with the intention of developing a miniaturized sensor prototype is the MSP430F2274, and both microcontrollers are similar, although the latter has small pin configuration (38 pins), with an optimal package style and does not need a thermal pad, which will ensure the possibility of miniaturization. In this specific software design, the MSP430 operates in two modes: ACTIVE Mode (270 μA at 1 MHz) and OFF Mode (RAM retention at 0.1 μA). The features presented allow the possibility of integrating new sensors in the future for other applications.

For communication purposes, the Chipcon CC2420 [19] is a true single-chip with 2.4 GHz IEEE 802.15.4 compliant RF transceiver, perfect for low power and low voltage wireless applications. This chip has a digital direct sequence spread spectrum baseband modem providing a gain of 9 dB and an effective data rate of 250 kbps. The antenna adopted seems to be a good option because it is intended for 2.4 GHz applications using Zigbee [6]. The size of the antenna is 6.1 mm × 3.9 mm × 1.1 mm, with a weight of 0.05 g.

**Figure 4.**
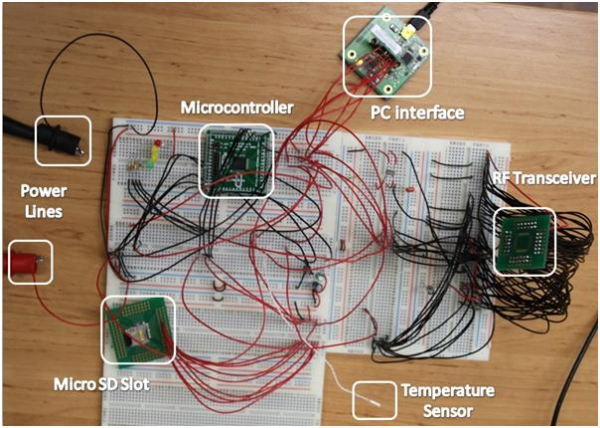
Physical testbed for performance evaluation and validation of the proposed temperature biosensor.

For a sensor node, in many applications and usages, it is highly relevant to have big data storage. Then, the memory size becomes a limitation because the microcontroller only has 1Kbyte RAM and 32 Kbyte Flash memory, which is not enough to record continuous data during a week. The major advantage of the sensor is providing an external microSD card with up to 2 Gbytes of memory. As may be seen in Figure 4, a power supply was used to power the testbed but for the final prototype already tested lithium batteries will be used.

For the design of the temperature sensor, the MA100 [24] thermistor was chosen. It is an NTC Type MA Biomedical Chip thermistor developed by GE Industrial Sensing and exclusively used for biomedical applications. Its main features fulfill the requirements of this solution. Its sensitivity ranges from 0 °C to 50 °C, and its size is 0.762 × 9.52 mm. The size, shape, temperature ranges, and its approval for medical applications were the major reasons for this choice. To get more accurate temperature readings and taking into account its goal, the temperature sensor must be placed inside the female cervix, which is an ideal thermal source to reach the core body temperature. For this proposed architecture, the MA100 is embedded in the mote platform.

Using Zigbee [7] communication, temperature monitoring can be performed in real-time mode. The sensor platform also saves measured values in the embedded microSD card and can send them to external devices within the Zigbee range area. This wireless communication is also used to transfer all the data stored in the sensor’s microSD card on demand.

Another important feature of this solution is mobility. After mote activation and correct placement inside the vagina, the woman can move freely and do whatever she wants with comfort. After switching on the data collection (operation performed through remote commands), the sensor starts measuring and continuously storing data in the microSD card. The monitored woman only has to take the sensor out when the date advised by the physician has been reached. During a long monitoring period, the sensor need only be removed for battery recharge.

For a regular medical observation, a physician can use the mote to measure the current core temperature of a patient in his office with a real-time connection to the sensor, as described above. On the other hand, he/she can connect to the sensor directly and retrieve all collected data to his computer. In both cases a physician can monitor and control the evolution of this biological parameter by observing a graphical representation of the measured values.

### 3.4. Temperature Sensor Integration

**Figure 5.**
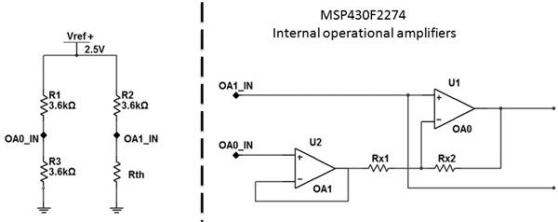
Thermistor signal acquisition circuit.

The integration of the temperature sensor into the small biosensor board was definitely the biggest challenge for this work, not excluding the board design. As mentioned above, the MA100 thermistor was the chosen temperature sensor, and some additional electrical equipment must be attached to this type of sensor (NTC) to ensure the linear behavior of the resistance. For an NTC thermistor a Wheatstone bridge to measure resistors and an operational amplifier are normally used to ensure this linear behavior. Due to the features of the microcontroller used in this design it is not necessary to add differential amplifiers because the microcontroller already has two configurable operational amplifiers (Figure 5). The medical applications of this biosensor must support core-body temperature variations around 0.1 °C, therefore, the calibration of the biosensor’s temperature sensor (MA100) is performed to a precision of 0.1 °C. The medical team that collaborates in this project validated this precision.

Equation (1) presents the relation between the two points on the Wheatstone bridge, which will generate the value of the differential tension on those two points (OAo_IN and OA1_IN). This value will be calculated internally by the microcontroller. It will be converted to a digital value on the available ADC10 line, considering the gain of the circuit amplifier. According to the proposed circuit some internal configurations need to be made to select the correct amplifier settings. As referred the microcontroller operational amplifiers can be configured with the OAFCx bits and to obtain a differential amplifier function it needs to be set to the “111” value. This mode allows internal routing of the OA signals for a two-opamp or three-opamp instrumentation amplifier. A two-opamp configuration with OA0 and OA1 will be used:

(1) $$V_{\mathrm{ADC}}=(\frac{R_{\mathrm{th}}}{R_{\mathrm{th}}+R_{2}})V_{\mathrm{ref}+}-(\frac{R_{3}}{R_{3}+R_{1}})V_{\mathrm{ref}+}$$

The analysis of temperature measurements is performed in off-line mode. To ensure good results, it is vitally important to know the exact time when each temperature measurement is taken. The biosensor only has a local time clock, which starts on biosensor start up. This clock cannot act as the real global time clock. To associate each measurement with the right global time clock instant, when a start command is sent to the biosensor, computer also sends its clock and date time (assuming the computer clock is global clock synchronized).

## 4. Results Analysis

**Figure 6.**
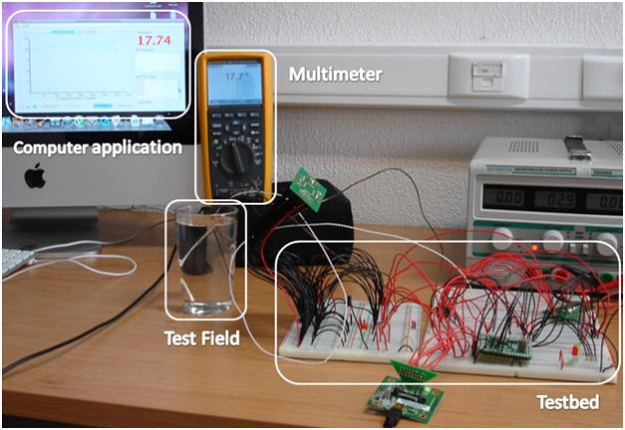
Experimental testbed for sensor validation of temperature measurements.

A temperature sensor was used to evaluate the proposed sensor design. Supported by the computer application presented in [15] temperature values were collected from the natural environment and from a glass of water. Several measurements were performed to make sure the sensor was recording the temperature values correctly or if any temperature variations were registered. To validate the sensor performance, the same measures were performed with a digital multi-function Fluke 289 [25] coupled with an 80BK-A thermocouple probe, as may be seen in Figure 6.

**Figure 7.**
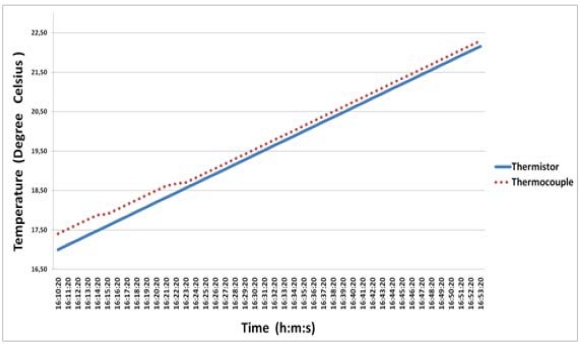
Performance comparison between temperature values acquired with a thermopar and a thermistor inside a glass of water.

The tests were performed in permanent contact with water due to the intended real environment application of the body-sensor (inside the vagina). The temperature of the water used in these tests was the natural one of water gathered directly from the pipes. The thermocouple probe and the thermistor have been placed together inside the glass of water without any other interference. As shown in Figure 7 the water temperature slowly increased over time due to the higher temperature of the room. In real time, it was possible to verify the collected values through the testbed with the temperature calibrator (Fluke 289) and the current on the thermistor terminals.

**Figure 8.**
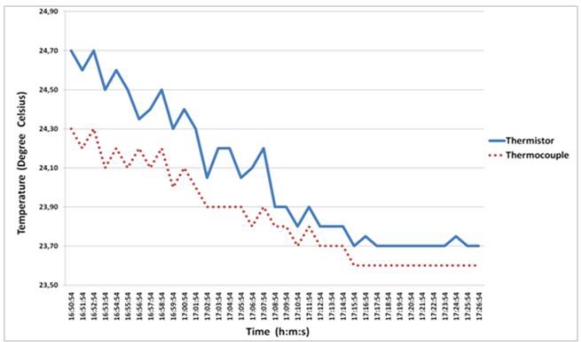
Performance comparison between temperature values acquired with a thermopar and a thermistor from a natural environment.

Another test was performed in order to confirm the accuracy of the temperature sensor, thus, a thermocouple probe and a thermistor have been used to record the ambient temperature of the room. Figure 8 presents the behavior of both temperature sensors and the difference between both temperature curves is clearly observed. In this test the thermistor presented a different behavior, having some difficulties in following or quickly reaching the same temperature value of the thermocouple probe. These variations registered by the thermistor could be due to the plastic enclosure that perhaps delays the quick response of the internal resistance in natural environments. According to these tests the behavior of the thermistor towards humidity in an environmental field and how it reacts to different variations of the temperature (increase/decrease) should be evaluated. These tests have contributed to the better calibration of the thermistor and allow us to conclude that thermistor is adequate for humidity fields.

## 5. Discussion and Conclusions

This paper presented the design of a new biosensor for e-health applications and the creation of a testbed to evaluate and validate the main functions of the new biosensor. This prototype was built to evaluate and validate the proposal, allowing the test of the several biosensor features. According to all the performed tests in a laboratory we can conclude that the temperature sensor acquires values with accuracy and it also collects and saves them on the microSD card correctly. Tests and performance comparisons were stressed in order to ensure the accuracy and usability of the biosensor. Those tests cannot be performed in (real) women due to health regulations/standards applicable to the use of electronic devices for human applications. According to the collected values it is possible to conclude that testbed was calibrated and measures the real environment temperature.
